# Footprints of innate immune activity during HIV-1 reservoir cell evolution in early-treated infection

**DOI:** 10.1084/jem.20241091

**Published:** 2024-10-28

**Authors:** Weiwei Sun, Ce Gao, Gregory Takashi Gladkov, Isabelle Roseto, Leah Carrere, Elizabeth M. Parsons, Carmen Gasca-Capote, John Frater, Sarah Fidler, Xu G. Yu, Mathias Lichterfeld, the RIVER Trial Study Group, Eric Sandström, Janet Darbyshire, Frank Post, Christopher Conlon, Jane Anderson, Mala Maini, Timothy Peto, Peter Sasieni, Veronica Miller, Ian Weller, Sarah Fidler, John Frater, Abdel Babiker, Wolfgang Stöhr, Sarah Pett, Lucy Dorrell, Matthew Pace, Natalia Olejniczak, Helen Brown, Nicola Robinson, Jakub Kopycinski, Hongbing Yang, Tomáš Hanke, Alison Crook, Stephen Kaye, Myra McClure, Otto Erlwein, Andrew Lovell, Maryam Khan, Michelle Gabriel, Rachel Bennett, Aminata Sy, Andrew Gregory, Fleur Hudson, Charlotte Russell, Gemma Wood, Hanna Box, Cherry Kingsley, Katie Topping, Andrew Lever, Mark Wills, Alex Fun, Mikaila Bandara, Damian Kelly, Simon Collins, Alex Markham, Mary Rauchenberger, Yinka Sowunmi, Shaadi Shidfar, Dominic Hague, Sarah Fidler, Sarah Pett, Mark Nelson, Maddalena Cerrone, Nadia Castrillo Martinez, Tristan Barber, Alexandra Schoolmeesters, Christine Weaver, Orla Thunder, Jane Rowlands, Christopher Higgs, Serge Fedele, Margherita Bracchi, Lervina Thomas, Peter Bourke, Nneka Nwokolo, Gaynor Lawrenson, Marzia Fiorino, Hinal Lukha, Sabine Kinloch, Margaret Johnson, Alice Nightingale, Nnenna Ngwu, Patrick Byrne, Zoe Cuthbertson, Martin Jones, Tina Fernandez, Aamanda Clarke, M. Fisher, Rebecca Gleig, Vittorio Trevitt, Colin Fitzpatrick, Tanya Adams, Fiounnouala Finnerty, John Thornhill, Heather Lewis, Kristin Kuldanek, Julie Fox, Julianne Lwanga, Hiromi Uzu, Ming Lee, Simon Merle, Patrick O’Rourke, Isabel Jendrulek, Taras ZarkoFlynn, Mark Taylor, Juan Manuel Tiraboschi, Tammy Murray

**Affiliations:** 1 Ragon Institute of MGH, MIT and Harvard, Cambridge, MA, USA; 2 Infectious Disease Division, Brigham and Women’s Hospital, Boston, MA, USA; 3 Nuffield Department of Medicine, University of Oxford, Oxford, UK; 4 Department of Infectious Disease, Imperial College and Imperial College NIHR Biomedical Research Centre, London, UK

## Abstract

Antiretroviral treatment (ART) initiation during the early stages of HIV-1 infection is associated with a higher probability of maintaining drug-free viral control during subsequent treatment interruptions, for reasons that remain unclear. Using samples from a randomized-controlled human clinical trial evaluating therapeutic HIV-1 vaccines, we here show that early ART commencement is frequently associated with accelerated and efficient selection of genome-intact HIV-1 proviruses in repressive chromatin locations during the first year after treatment initiation. This selection process was unaffected by vaccine-induced HIV-1-specific T cell responses. Single-cell proteogenomic profiling demonstrated that cells harboring intact HIV-1 displayed a discrete phenotypic signature of immune selection by innate immune responses, characterized by a slight but significant upregulation of HLA-C, HLA-G, the IL-10 receptor, and other markers involved in innate immune regulation. Together, these results suggest an accelerated immune selection of viral reservoir cells during early-treated HIV-1 infection that seems at least partially driven by innate immune responses.

## Teaser

This study demonstrates that early initiation of ART enhances immune selection pressure on HIV-1 reservoir cells and accelerates the accumulation of intact proviruses in heterochromatin regions; phenotypic profiling of reservoir cells suggests that these effects are primarily driven by innate immunity.

## Introduction

The life-long persistence of HIV-1 infection despite highly efficient antiretroviral therapy (ART) is due to small numbers of CD4 T cells that harbor chromosomally integrated, replication-competent viral DNA and are not susceptible to currently-available antiviral drugs (Finzi et al., 1997; Wong et al., 1997). These cells, frequently referred to as “HIV-1 reservoir cells,” are highly durable, can effectively avoid host immune activity, and drive rebound viremia after treatment interruptions. A number of studies have demonstrated that seeding of HIV-1 reservoir cells occurs during the early stages of viral infection (Bruner et al., 2016; Chun et al., 1998; Lee et al., 2019), presumably within the first days after viral transmission, and cannot be prevented or blocked through early antiviral treatment initiation (Henrich et al., 2017). Nevertheless, treatment commencement during acute infection can be associated with a weaker ability of viral reservoir cells to drive viral rebound during subsequent treatment interruptions (Saez-Cirion et al., 2013), and some (Fidler et al., 2017; Gossez et al., 2019; Namazi et al., 2018; Passaes et al., 2024; Rosenberg et al., 2000), although not all (Colby et al., 2018), studies in humans or non-human primates indicated higher probabilities of maintaining post-treatment drug-free control when treatment is started early; this suggests that early treatment initiation may modulate viral reservoir cell establishment in favor of the host.

Prior work demonstrated that viral reservoir cells decay faster when antiretroviral treatment is initiated soon after viral transmission, likely due to host immune responses that may be more effective in eliminating virally-infected cells during the early stages of viral infection (Buzon et al., 2014; Massanella et al., 2021). However, how antiviral immune responses can interface with, engage, and target viral reservoir cells following early antiviral treatment initiation is mostly unclear. Of note, HIV-1 reservoir cells were, for a long time, regarded as being almost entirely protected from antiviral immune responses due to very low or absent viral transcription, a condition for which the term “HIV-1 latency” has been coined (Ruelas and Greene, 2013). However, several recent studies have challenged this view and suggest that a remarkable fraction of reservoir cells can actively transcribe viral genes (Collora et al., 2022; Einkauf et al., 2022; Yukl et al., 2018), consistent with the notion that current antiretroviral agents do not inhibit HIV-1 gene expression. The majority of proviruses producing such viral transcripts are integrated into accessible chromatin and appear to be longitudinally selected against, while genome-intact proviruses integrated into heterochromatin regions seem to have a selection advantage and persist or expand over time (Einkauf et al., 2022). This observation is most readily explained by immunological targeting of infected cells harboring transcriptionally active proviruses integrated into accessible euchromatin that, putatively, are more easily recognized by immune sensors. However, this selection process is typically slow and requires up to two decades of continuous suppressive therapy to become visible with current analysis techniques (Lian et al., 2023). When ART is initiated during acute infection, viral reservoir cell seeding coincides with the strong initial immune response to HIV-1 that involves a massive “cytokine storm” with systemic release of pro-inflammatory cytokines and activation of innate immune cells (Stacey et al., 2009). Adaptive antiviral T cell responses emerge within several weeks after infection and are typically narrowly focused toward a few immunodominant epitopes; these immune responses can have strong antiviral activities and may contribute to the decline of high-level viremia to the viral set point during untreated primary infection (Koup et al., 1994; Ndhlovu et al., 2019). How the specific immune milieu and microenvironment during acute infection shape the viral reservoir cell pool when treatment is initiated early is currently unknown.

The influence of antiviral immune responses on HIV-1 reservoir cell evolution and persistence was formally analyzed in the RIVER study (Fidler et al., 2020), a randomized-controlled human clinical trial involving individuals who were identified and started on ART in acute/early HIV-1 infection. Study participants were randomized to receive an AdV63-vectored immunogen incorporating conserved viral epitopes (HIV.Consv) (Letourneau et al., 2007), followed by an modified vaccinia Ankara (MVA)-vectored vaccine booster and 10 consecutive oral doses of vorinostat, a moderately potent histone deacetylase inhibitor designed to reverse viral latency and induce viral gene transcription (Archin et al., 2012). Alternatively, study participants were randomized to a control group not receiving any medical interventions apart from ART. Although this study reported a statistically significant increase in HIV-1-specific T cell responses in the vaccination group as a result of therapeutic immunization, there were no differences between the two study groups with regard to frequencies of total proviral DNA copies (determined by quantitative PCR assays evaluating HIV-1 gag sequences) and to numbers of replication-competent proviruses identified using in vitro viral outgrowth assays, a technology that can identify ∼1–2% of all intact proviruses (Ho et al., 2013). Moreover, the administration of vorinostat in the vaccination group failed to induce significant increases in proviral transcription in this study.

Using samples from this study, we here evaluated the evolution of intact and defective proviruses during the first 1–2 years of ART initiation in participants from both study groups. Our data demonstrated that early treatment initiation during acute HIV-1 infection can be associated with rapid and effective selection of intact proviruses in heterochromatin regions; however, this selection process was unrelated to vaccine-induced HIV-1-specific T cell responses. Single-cell proteogenomic profiling revealed that cells harboring intact proviruses during acute infection frequently displayed significant upregulation of phenotypic markers involved in innate immunity, including HLA-C, HLA-G, and the receptors for IL-10 and the TGF-β, suggesting that innate immune mechanisms may play a role for immune selection of HIV-1 reservoir cells during acute HIV-1 infection.

## Results

### Quantitative evaluation of intact HIV-1 proviruses following early ART initiation

**Figure 1.**
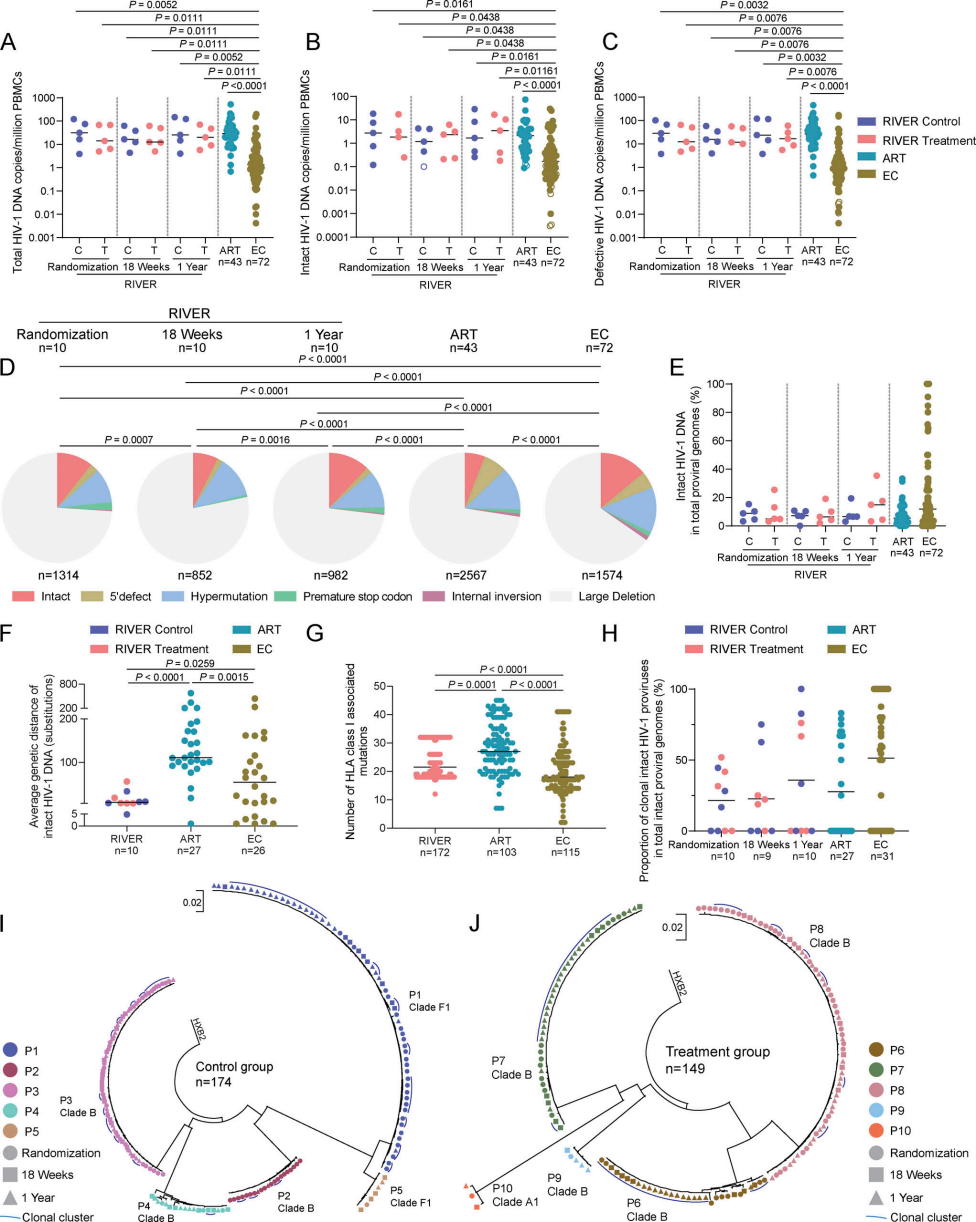
**Proviral reservoir profile in RIVER study. (A–C)** Frequencies of total (A), intact (B), and defective (C) HIV-1 proviruses in PBMCs from the control and treatment groups of the RIVER study at indicated time points. Data from people treated with antiviral therapy for a median of 9 years (ART) and from ECs are shown for comparison. Open circles indicate data at the limit of detection. Horizontal bars indicate the median. FDR-adjusted two-sided Kruskal–Wallis nonparametric tests were used for statistical comparisons relative to EC and ART cohorts. Only significant P values (P < 0.05) are presented. C: RIVER control group; T: RIVER treatment group. **(D)** Pie charts reflect the composition of HIV-1 DNA sequences from RIVER participants at indicated time points. Data from ART and EC cohorts are shown for reference. FDR-adjusted chi-square tests were used for statistical analysis. Numbers of participants are listed for each study group; numbers of proviral sequences are depicted below pie charts. **(E)** Proportions of intact HIV-1 DNA copies in total proviral genomes from the control and treatment groups of the RIVER study at randomization, 18 wk, and 1 year time points. Data from ART and EC cohorts are shown for comparison. Horizontal bars indicate the median and *n* represents the number of study participants. C: RIVER control group; T: RIVER treatment group. **(F)** The average genetic distance of intact proviruses in each participant from RIVER, ART, and EC cohorts was determined by pair-wise comparisons between all unique intact proviruses within a given study participant. FDR-adjusted two-sided Kruskal–Wallis nonparametric test was used for statistical analysis. Horizontal bars indicate the median, and *n* represents the number of study participants in whom at least two distinct intact proviruses were detected. **(G)** Number of HLA class I associated mutations within intact proviral sequences from RIVER, ART, and EC cohorts, determined by an algorithm described by Carlson et al. (2012). Each dot represents one intact provirus. Only clade B sequences were included, and clonal sequences were counted once. FDR-adjusted two-sided Kruskal–Wallis nonparametric test was used. Horizontal bars indicate the median, and *n* represents the number of intact sequences from each cohort. **(H)** Proportions of clonal intact HIV-1 proviruses (defined as proviral sequences detected at least two times) within total intact HIV-1 proviruses from RIVER participants at indicated time points, and in ART and EC reference cohorts. Horizontal bars indicate the mean and *n* represents the number of study participants. All individuals with at least two detectable intact proviral sequences were included. **(I and J)** Circular maximum likelihood phylogenetic trees of intact HIV-1 proviruses from RIVER control group (I) and RIVER treatment group (J). Participant (P) and clade information were indicated. Each symbol represents one intact provirus. Color coding reflects different participants. *n* represents the number of intact proviral sequences from each group. Symbols indicate different time points: dot: Randomization time point; square: 18 wk; triangle: 1-year time point; HXB2, HIV-1 reference sequence. **(A–H)** P values are listed when considered significant (P < 0.05) after adjustment for multiple comparison testing.

**Figure S1.**
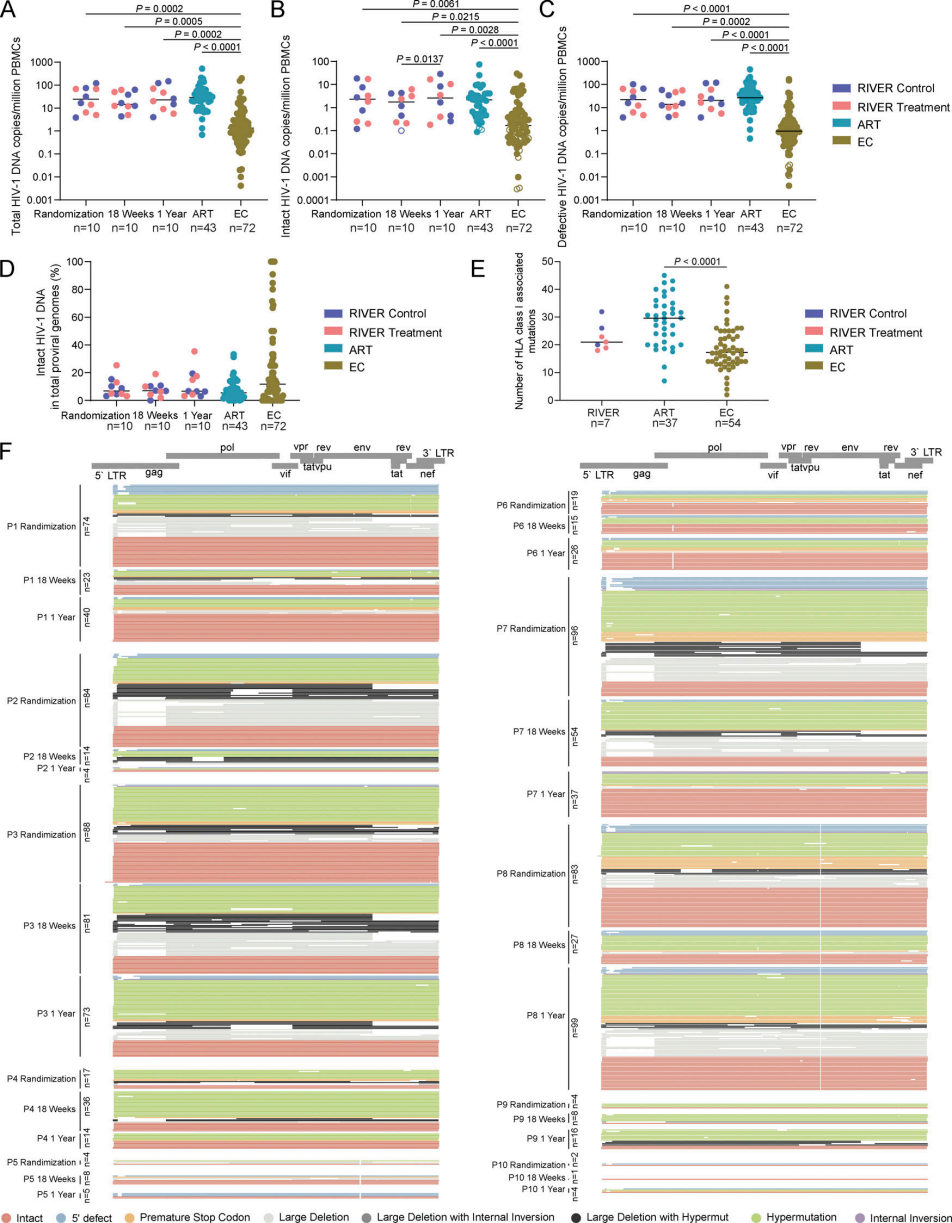
**Detailed analysis of HIV-1 DNA sequences from RIVER participants. (A–C)** Frequencies of total (A), intact (B), and defective (C) HIV-1 proviruses in PBMCs from all RIVER study participants at indicated time points. Data from people treated with antiviral therapy for a median of 9 years (ART) and from ECs are shown for comparison. Open circles indicate data at the limit of detection. Horizontal bars indicate the median. FDR-adjusted two-sided Kruskal–Wallis nonparametric tests were used for statistical comparisons relative to EC. Wilcoxon matched-pairs signed rank test was used to compare data from RIVER participants at different time points. *n* represents the number of study participants. **(D)** Proportions of intact HIV-1 DNA copies in total proviral genomes from all RIVER study participants at randomization, 18 wk, and 1 year time points. Data from ART and EC cohorts are shown for comparison. Horizontal bars indicate the median and *n* represents the number of study participants. **(E)** Number of HLA class I–associated mutations within intact proviral sequences from RIVER control and treatment groups, ART and EC cohorts, determined by an algorithm described by Carlson et al. (2012). Each dot represents averaged data from one study participant. Only clade B sequences were included, and clonal sequences were counted once. FDR-adjusted two-sided Kruskal–Wallis nonparametric test was used. Horizontal bars indicate the median and *n* represents the number of participants from each cohort. **(F)** Virograms highlighting the proviral reservoir profile in RIVER study participants. Each horizontal line reflects one HIV-1 DNA sequence. Color-coding reflects classification of proviral sequences as intact or defective. Sample time points are indicated on the y-axis.

We longitudinally analyzed the evolution of intact proviruses in a total of 10 participants from the RIVER study who initiated ART during acute/early HIV-1 infection; five of the study persons were in the control group and the five remaining persons had been randomized to receive the therapeutic vaccines and vorinostat. Cryopreserved peripheral blood mononuclear cells (PBMC) samples from three different time points were analyzed in each study participant: One sample was collected at the time of randomization when most study participants had received ∼6 mo of suppressive ART; one sample was collected 18 wk later, at the time when the administration of study drugs was completed and individuals had reached the primary study endpoint; and one sample collected during a follow-up visit ∼1 year after the randomization time point. Clinical, demographical, and immunogenetic characteristics of the study participants are shown in Table S1. As an initial analysis step, we performed single-genome, near full-length amplification of individual proviral species, followed by next-generation sequencing of selected (near full-length) amplicons and biocomputational identification of genome-intact proviruses lacking lethal sequence variations. As a modification to this protocol, DNA diluted to single proviral genomes was first subjected to whole-genome amplification by phi29-based multiple displacement amplification (MDA), permitting subsequent combined assessments of proviral sequences and corresponding chromosomal integration sites. These methods, termed full-length individual proviral sequencing (FLIP-Seq) (Lee et al., 2017) and matched integration site and proviral sequencing protocol (MIP-Seq) (Einkauf et al., 2019) in our prior work, produced a total of *n* = 3,148 individual proviral amplification products from all 10 RIVER study participants combined, of which *n* = 1,056 were sequenced and *n* = 323 were classified as genome-intact, using a previously described computational pipeline (Jiang et al., 2020). We observed that the frequencies of total, intact, and defective proviruses remained relatively stable during the analysis time period and did not differ significantly between individuals randomized to the control group versus the treatment group (Fig. 1, A–C and Fig. S1, A–F). Of note, the frequencies of total, intact, and defective proviral sequences of the RIVER study participants, regardless of randomization outcomes, were higher than those in a reference cohort of elite controllers (ECs) described in our prior work (Jiang et al., 2020), but did not differ significantly from a group of individuals who had started treatment in chronic infection and remained on suppressive ART for a median duration of 9 years (Table S1) (range 1–22 years) (Fig. 1, A–C and Fig. S1, A–C). The majority of proviral sequences isolated from both arms of the RIVER study harbored large deletions or other lethal sequence mutations; the proportions of genome-intact proviruses among all detected proviruses reached 10.73% at randomization, 7.39% at 18 wk to 12.12% at the 1-year time point (Fig. 1 D and Fig. S1 D) and did not differ notably between the control group and the treatment group (Fig. 1 E). The degree of intra-individual proviral diversity, determined by pair-wise comparisons between individual intact proviruses within a given study person, was remarkably low in RIVER study participants, and did not differ between individuals randomized to the control versus treatment groups (Fig. 1 F); however, intra-individual sequence diversity in RIVER participants was significantly smaller relative to the reference cohorts of ART-treated persons and ECs (Fig. 1 F). Notably, in the RIVER study participants, we observed relatively low frequencies of viral sequence polymorphisms associated with autologous HLA class I alleles, consistent with a limited degree of adaptation to HLA class I–dependent immune pressure in this study cohort, likely due to early treatment initiation (Fig. 1 G and Fig. S1 E). Importantly, phylogenetic studies demonstrated that clonal HIV-1 proviruses, characterized by identical proviral sequences and/or integration sites, were already observed at the earliest analysis time point in some RIVER study participants (Fig. 1, H–J). The relative proportions of intact clonal sequences among the total pool of intact proviral genomes tended to increase over time, irrespective of randomization outcomes (Fig. 1 H); however, these changes did not reach statistical significance. Of note, we failed to detect statistical associations between the frequencies of total or intact proviruses and corresponding frequencies of HIV-1-specific CD4 and CD8 T cells determined by intracellular cytokine staining of IFN-γ, IL-2, TNF-α, CD154, and CD107a, according to a protocol described in a previous manuscript (Fidler et al., 2020) (Table S2). Together, these results suggest that treatment initiation during acute HIV-1 infection induces a reservoir profile that is low in phylogenetic complexity and involves clusters of clonally expanded HIV-1 reservoir cells that can be detected as early as 6 mo after treatment initiation; however, the quantitative longitudinal evolution of the reservoir cell pool during primary infection seems unaffected by naturally-occurring and vaccine-induced adaptive T cell responses to HIV-1.

### Evolution of proviral integration sites

**Figure 2.**
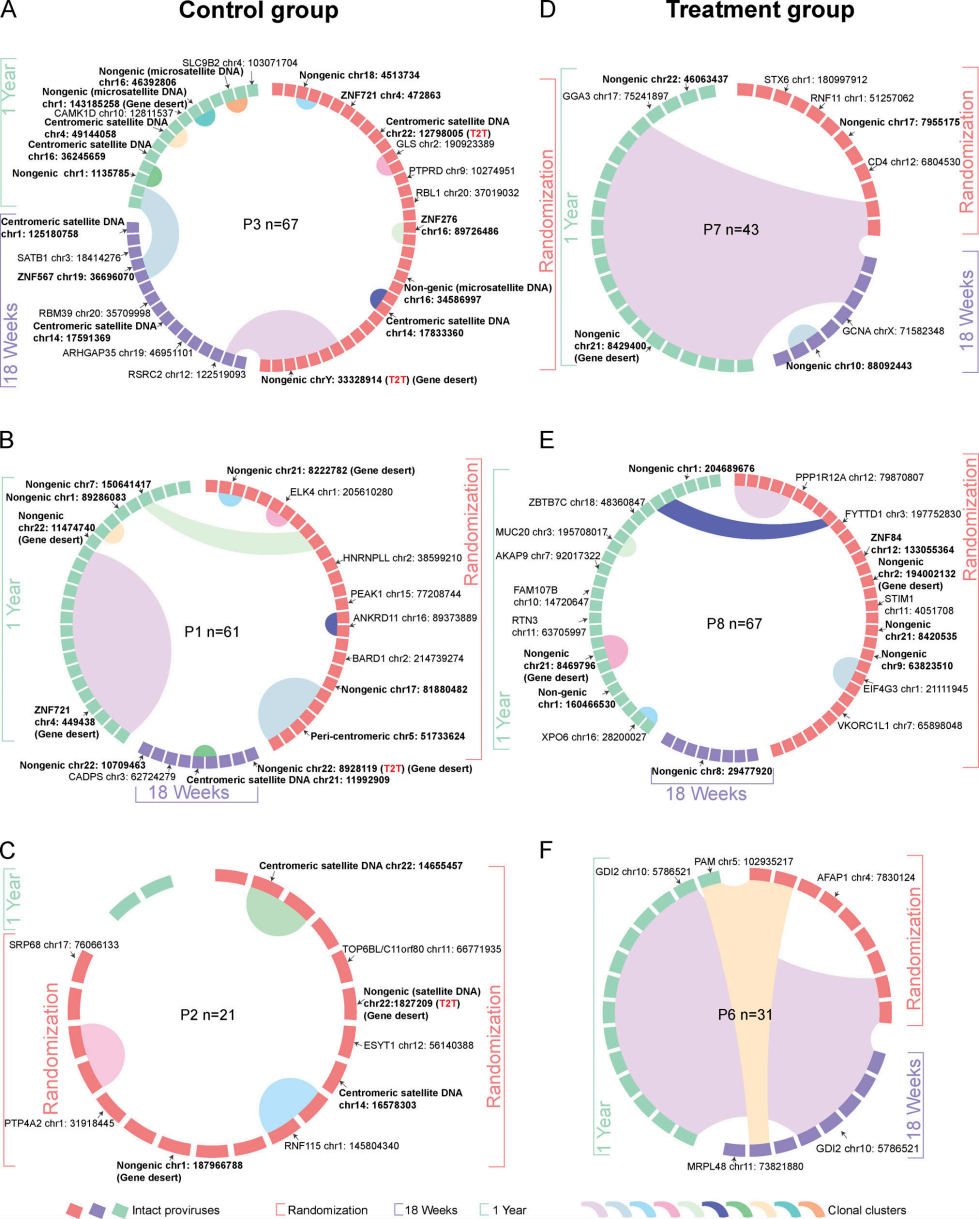
**Integration site landscape of intact proviruses in RIVER study participants. (A–F)** Circos plots reflecting the clonality and chromosomal locations of all intact proviral sequences isolated at indicated time points in each participant (A–C: RIVER control group; D–F: RIVER treatment group). Genomic coordinates are generally indicated in the Hg38 human reference genome nomenclature; in selected cases, coordinates in the T2T reference are indicated if integration sites could not be mapped to the Hg38 genome. Each symbol reflects one intact provirus. Color coding of the symbol and the bracket around the plot reflect indicated time points. Clonal sequences, defined by complete sequence identity and/or identical integration sites, are highlighted. P: participant. Integration sites annotated by the T2T reference genome are indicated. *n* represents the number of intact proviral sequences from each participant.

The location of individual proviruses within the human genome can have a profound impact on proviral transcriptional activity and may influence the vulnerability of proviruses to host immune recognition. To profile changes in chromosomal integration sites of defined proviral species, we used integration site loop amplification (ISLA) assays (Wagner et al., 2014) to assess the chromosomal positions of intact proviruses after whole-genome amplification of single proviruses, using our previously described MIP-Seq protocol (Einkauf et al., 2019). Overall, we analyzed a total of *n* = 2,510 individual proviruses with the MIP-Seq assay; a total of *n* = 2,273 of these sequences were defective, while *n* = 237 sequences were classified as genome-intact. In six RIVER study participants, the frequencies of intact proviruses were sufficiently high to perform a high-resolution longitudinal analysis of their respective integration sites from the available numbers of PBMC (Fig. 2, A–F). In several of these study participants, we noted that the integration site profile of intact proviruses deviated from the typical genic locations that are preferred by the viral integration machinery (Schroder et al., 2002) and that dominate the integration site landscape of intact proviruses in persons with moderate durations of ART (∼10 years) initiated during chronic infection (Einkauf et al., 2019). Instead, chromosomal locations of intact proviruses in these study persons were enriched for genomic positions with heterochromatin features (Fig. 2, A–F). This was particularly obvious for study participants P1 and P3 (both randomized to the control group) and in study person P7 (randomized to the treatment group); in these individuals, we frequently noted clonal and non-clonal intact proviruses that were integrated into centromeric or peri-centromeric satellite DNA, in gene deserts, and in ZNF genes on chromosome 4, 16, and 19. Persistence of intact proviruses in non-genic regions and centromeric satellite DNA were also observed in study person P2 (randomized to the control group) and in P8 (randomized to the treatment group), although selection of intact proviruses in repressive chromatin was more limited in these individuals. Of note, proviral persistence in heterochromatin locations was previously primarily detected in ECs (Jiang et al., 2020), in post-treatment controllers (Lian et al., 2023), and in individuals after long-term ART (Huang et al., 2021; Lian et al., 2023), and can be interpreted as a result of immune selection pressure that preferentially eliminates proviruses in more accessible chromatin regions with increased permissiveness to viral transcription. Since heterochromatin regions are insufficiently captured in the human reference genome Hg38 that was generated with short-read sequencing techniques (Schneider et al., 2017), we also aligned hybrid next-generation sequence reads spanning viral–host junctions to the recently described telomere-to-telomere genome (T2T) (Nurk et al., 2022). The T2T genome was assembled from long-read sequences of the haploid cell line CHM13 derived from a hydatidiform mole; the absence of allelic variation in the haploid sequence permitted unambiguous sequence resolution in this genome, specifically in non-genic regions containing highly repetitive genetic elements. Alignment to the T2T genome identified several integration sites of intact proviruses in the acrocentric chromosomes 22 and Y (in study persons P1, P2, and P3) that were missed when integration site identification was solely based on the Hg38 reference genome, suggesting that integration and persistence of intact proviruses in these chromosomal regions may be more common than previously assumed. Interestingly, in one “outlier” study person (P6, randomized to the treatment group), we could not detect the persistence of intact proviruses in heterochromatin regions; instead, this study person displayed a large clone of intact proviruses integrated in the gene GDI2, known to be involved in G protein–dependent signal transduction and associated with autonomous cell proliferation and malignant cell transformation (Liu et al., 2021). Clonal proliferation of HIV-1-infected CD4 T cells harboring proviruses integrated into proliferation- or cancer-associated genes has been documented before and may reflect accelerated cell growth resulting from retroviral insertional mutagenesis (Liu et al., 2020; Wagner et al., 2014).

**Figure 3.**
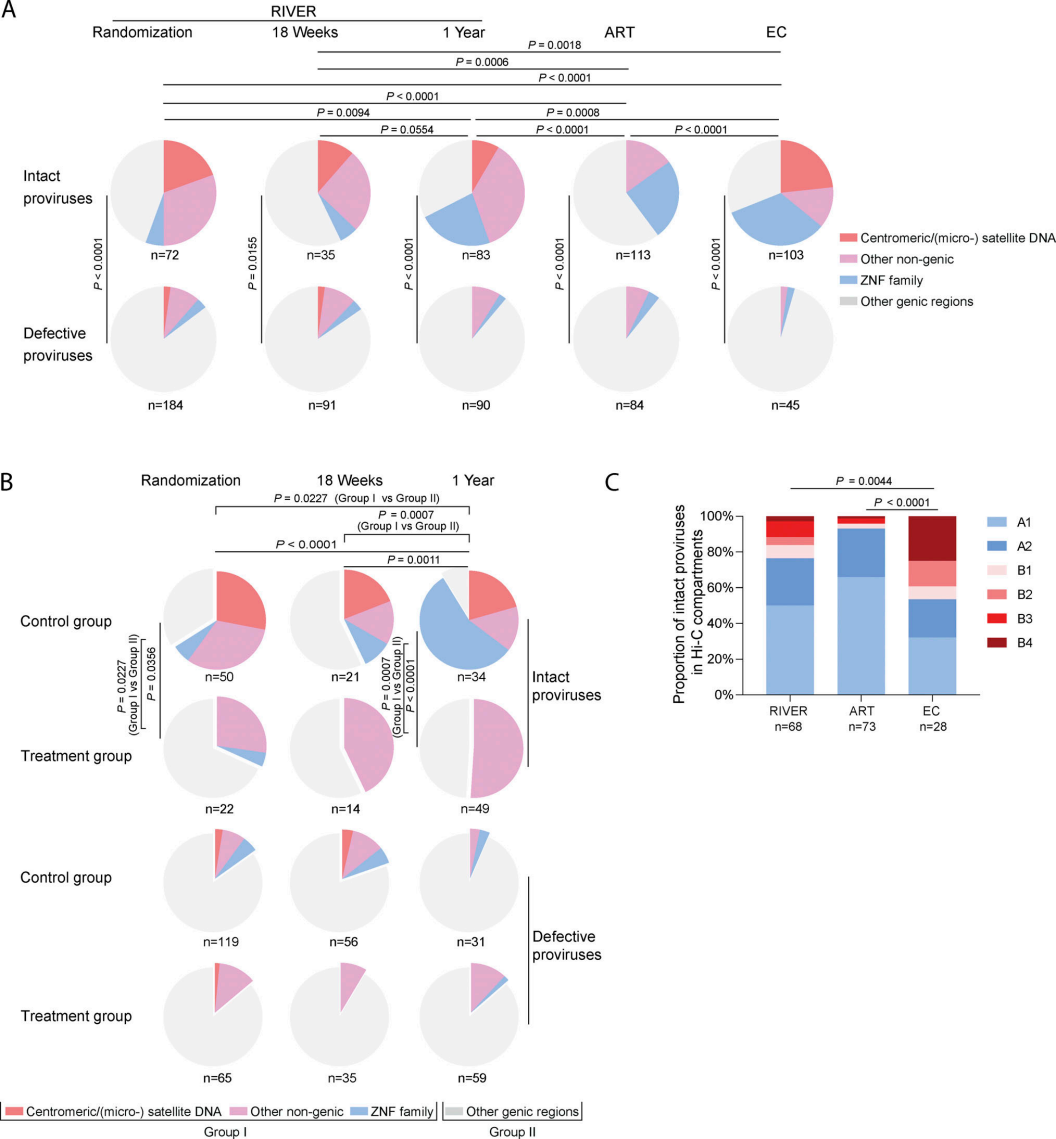
**Genomic and epigenetic integration site features of intact proviruses. (A)** Pie charts reflect the proportions of intact (upper panel) and defective (lower panel) proviruses within defined genomic regions at indicated time points of the RIVER study, and in ART and EC reference cohorts. Clonal sequences were counted individually, and *n* represents the number of integration sites. FDR-adjusted chi-square tests were used for statistical analysis. **(B)** Pie charts reflect the proportions of intact (upper panels) and defective proviruses (lower panels) within defined genomic regions at indicated time points of the control group and treatment group of the RIVER study. Clonal sequences were counted individually, and *n* represents the number of integration sites. FDR-adjusted chi-square tests were used to compare each pie chart. Comparisons of proviruses in genomic regions with “block and lock” heterochromatin features (group I) and corresponding proportions in genic locations (group II) between individual pie charts were analyzed using FDR-adjusted Fisher’s exact tests. **(C)** Proportions of intact proviruses located in structural compartments/subcompartments A (transcriptionally-active chromatin) and B (transcriptionally repressed chromatin) are shown, as determined by alignment of integration site coordinates to Hi-C seq data (Rao et al., 2014). Integration sites not covered in the reference dataset were excluded. Clonal sequences with the same integration sites were counted once. FDR-adjusted two-sided Kruskal–Wallis nonparametric test was used. *n* represents the number of unique integration sites from each cohort.

**Figure S2.**
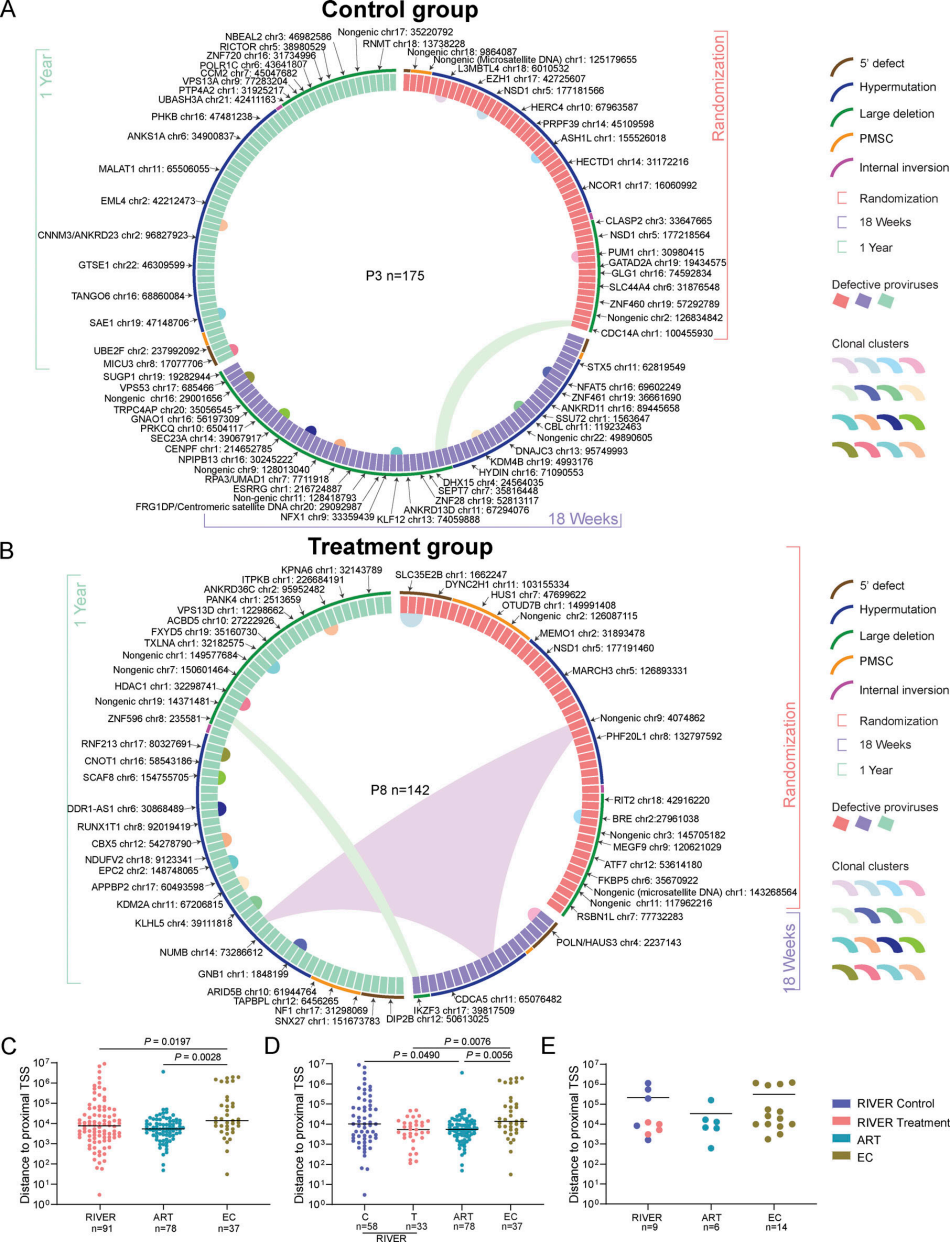
**Comparative analysis of HIV-1 DNA sequences isolated from RIVER study participants. (A and B)** Circos plot reflecting the clonality and chromosomal locations of all defective proviral sequences isolated at indicated time points in participant 3 from RIVER control group (A), and participant 8 from RIVER treatment group. Each symbol reflects one provirus. Color coding of symbol and the bracket around the plot reflect indicated time points. Clonal sequences, defined by complete sequence identity and/or identical integration sites, are highlighted. *n* represents the number of defective HIV-1 proviral sequences. P: participant. Color-coded arches around the plots indicate types of proviral sequences. **(C–E)** Chromosomal distance between integration sites of intact proviruses to most proximal host TSSs listed in the genome browser. Clonal sequences with the same integration sites were counted once. FDR-adjusted two-sided Kruskal–Wallis nonparametric test was used. **(C and D)** Horizontal bars indicate the median, and *n* represents the number of unique integration sites from each cohort (C) and from each randomization arm (D). **(E)** Average distance of integration sites of intact proviruses to most proximal TSS in each study participant. Horizontal bars indicate the mean, and *n* represents the number of participants from each cohort.

**Figure 4.**
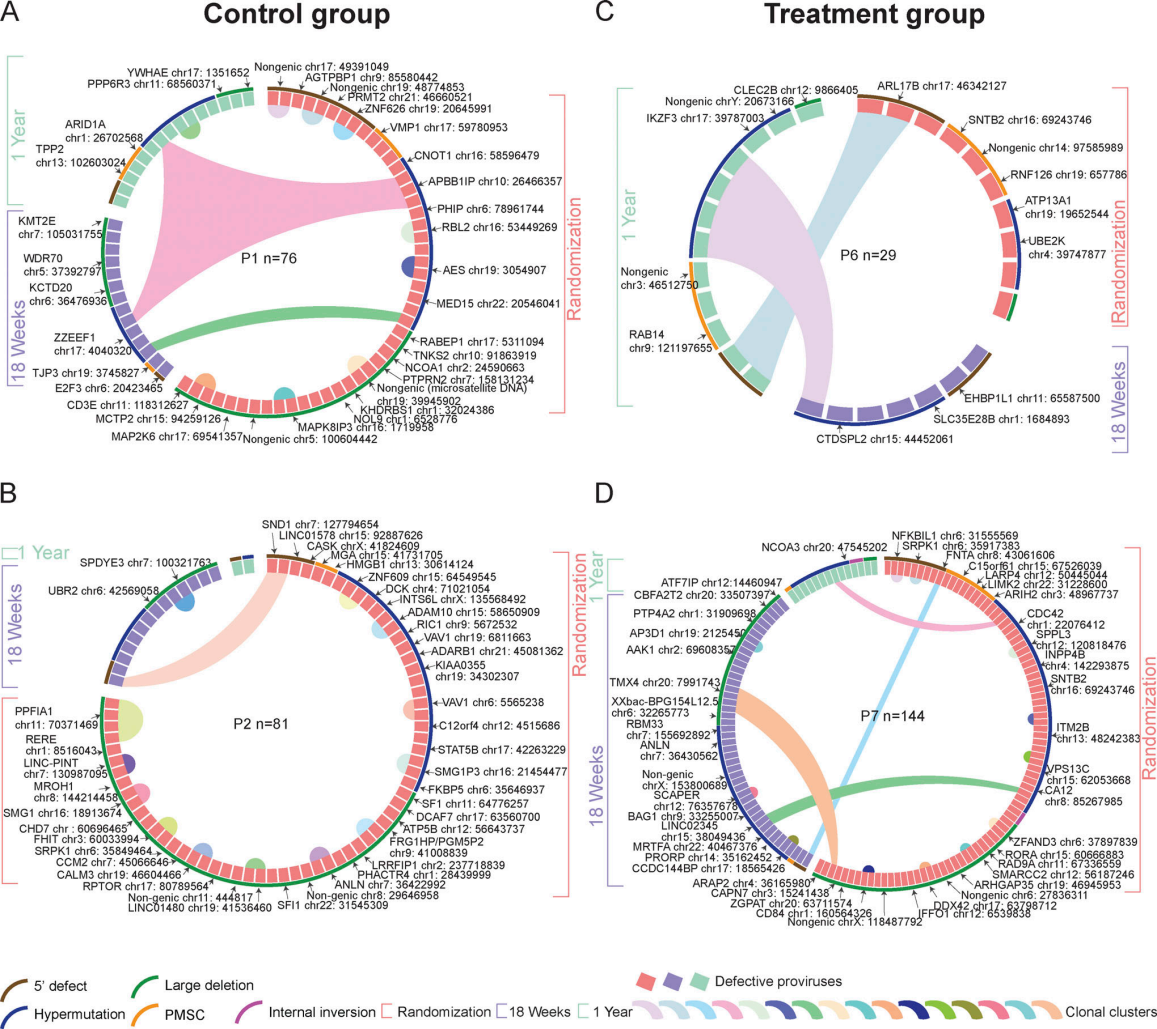
**Integration sites of defective HIV-1 proviruses in the RIVER study. (A–D)** Circos plots reflecting the clonality and chromosomal locations of all defective proviral sequences isolated at indicated time points in participants 1 (A), 2 (B), 6 (C), and 7 (D). A and B: RIVER control group; C and D: RIVER treatment group. Each symbol represents one defective provirus. Color coding of the symbol and the bracket around the plot reflect indicated time points. Clonal sequences, defined by complete sequence identity and/or identical integration sites, are highlighted. *n* represents the number of defective HIV-1 proviral sequences from each participant. P: participant. Color-coded arches around the plots indicate types of proviral sequences.

In total, we observed that in all analyzed RIVER study participants combined, intact proviruses integrated in satellite DNA, ZNF genes, and non-genic regions accounted for 55.56% of all integration sites of intact proviruses at randomization. At week 18, the time of the primary endpoint, the proportion of intact proviruses in heterochromatin regions was slightly lower (42.86%); this may be due to lower numbers of cells available for investigation from this time point and because the frequency of proviruses sampled at that time was more limited. Notably, after 1 year of follow-up, the proportion of intact proviruses in these atypical chromosomal locations had increased to 67.47% and approximated the corresponding proportion of intact proviruses in EC (68.93%) (Fig. 3, A and B), suggesting an accelerated selection of intact proviruses in repressive chromatin locations within the first year after early ART initiation. Correspondingly, there was a slight, non-significant trend for increased chromosomal distances of intact proviruses to the most proximal transcriptional start site (TSS) in RIVER study participants relative to individuals who had started treatment in chronic infection and remained on treatment for ∼9 years (Fig. S2, C–E). We also noted that the distribution of intact proviruses in chromosomal 3D compartments, determined based on an alignment of proviral integration site coordinates to previously described Hi-C data (Rao et al., 2014) was more biased toward heterochromatin compartments in RIVER study participants compared with individuals who started ART in chronic infection (Fig. 3 C). However, the selection of intact proviruses in heterochromatin 3D compartments and enhanced distance to host TSSs in RIVER participants was less pronounced when compared with the EC cohort (Fig. 3 C and Fig. S2, C–E). Of note, we failed to detect a statistical association between proportions of intact proviruses integrated in repressive chromatin and the corresponding proportions of autologous mono-, pauci-, or poly-functional HIV-1-specific CD4 or CD8 T cell responses (Table S4); in fact, the relative abundance of intact proviruses located in heterochromatin regions in the control group of the RIVER study exceeded corresponding numbers in the treatment group at baseline and at the 1-year follow-up time point (Fig. 3 B), likely as a result of stochastic effects in the randomization process in the relatively low number of our study participants. These results suggest that vaccine-induced HIV-1-specific T cells are less likely to play a role in selecting intact proviruses in heterochromatin locations, at least during the early stages of viral infection. Importantly, an extensive effort to characterize integration sites of defective proviruses demonstrated no specific selection of viral sequences in repressive chromatin locations (Fig. 3, A and B; Fig. 4, A–D; and Fig. S2, A and B); this observation indicates that our integration site detection technology is not intrinsically biased toward heterochromatin positions and suggests that defective proviruses are less vulnerable to host immune selection forces. Together, these results suggest the accelerated and efficient selection of intact proviruses in heterochromatin regions during early-treated HIV-1 infection; this selection process seems independent of HIV-1-specific T cell responses.

### Phenotypic profile of HIV-1 reservoir cells after early ART initiation

**Figure 5.**
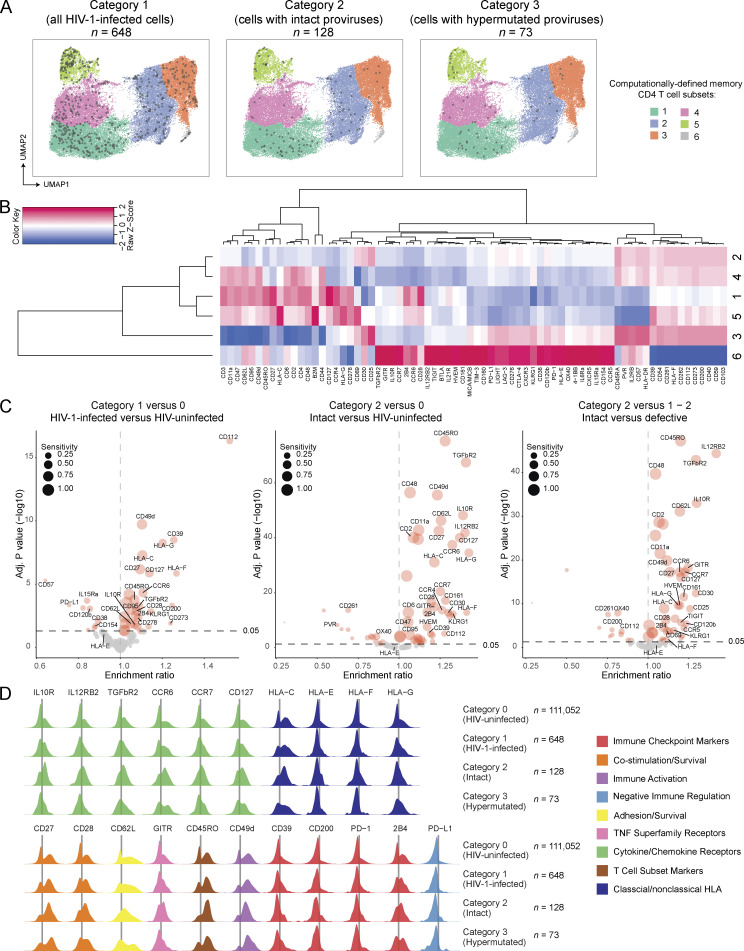
**Phenotypic characteristics of HIV-1 reservoir cells in the RIVER study. (A)** 2D UMAP diagrams reflecting the global phenotypic profile of HIV-1 reservoir cells in memory CD4 T cells isolated from peripheral blood of three RIVER study participants. Six computationally defined spherical clusters are indicated. One plot is shown each for category 1, category 2, and category 3 cells. **(B)** Heatmap representing the normalized phenotypic profile of cells in each spherical cluster, based on 72 surface markers included in this study. **(C)** Volcano plots reflecting the enrichment ratio of marker-positive cells and corresponding FDR-adjusted (adj.) P values for all 72 surface markers were included in this study. Selected markers were labeled individually. Marker sensitivities, defined as the proportions of marker-positives cells in the indicated categories of cells, were indicated by dot sizes. Comparisons between indicated categories of cells are depicted; bootstrapped data from all three participants are shown. Significance was tested using a two-sided chi-squared test; FDR-adjusted P values are shown. **(D)** Density plots reflecting the expression of selected surface markers on indicated categories of HIV-1-infected cells from the three RIVER study participants.

**Figure S3.**
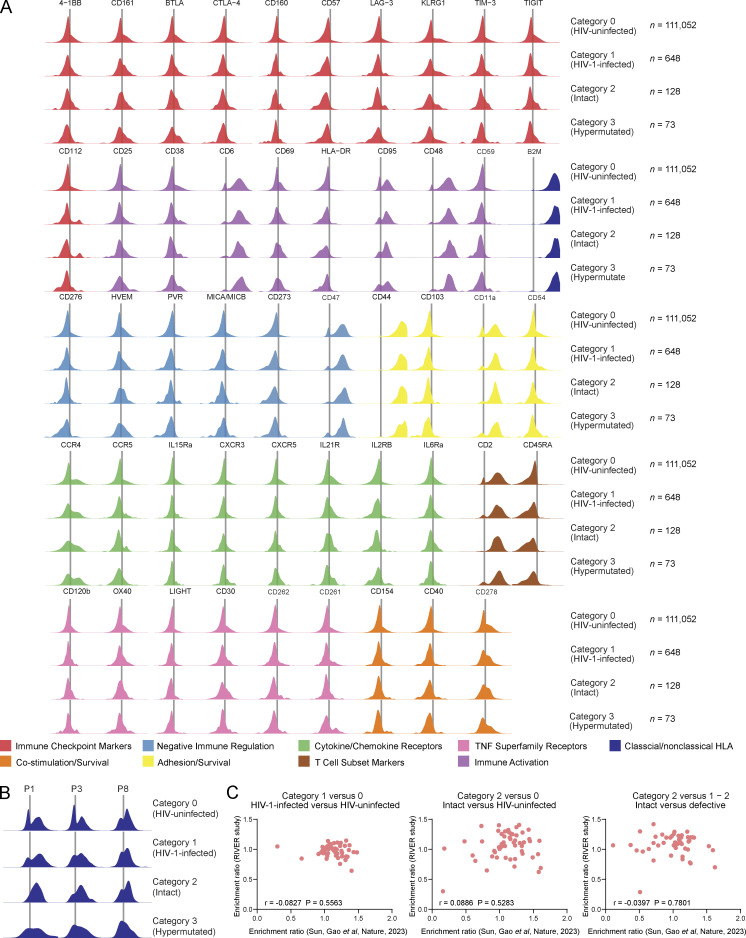
**Comprehensive analysis of phenotypic characteristics of HIV-1 reservoir cells. (A)** Density plots reflecting the expression of remaining surface markers on indicated categories of cells from three participants. Category 0 cells are shown as a reference. **(B)** Histograms reflecting expression of HLA-C on carriers of indicated HLA-C alleles. **(C)** Correlation between enrichment ratios of phenotypic markers on HIV-1 reservoir cells in the RIVER study and in a prior study by Sun et al. (2023). Correlations were analyzed by Spearman correlation coefficients.

To explore and infer immune mechanisms that may contribute to the selection of HIV-1 reservoir cells during early-treated viral infection, we used a recently developed proteogenomic profiling technique, termed PheP-Seq (phenotypic and proviral sequencing) in our prior work (Sun et al., 2023). For this analysis, we selected three study participants (two from the control group [P1, P3] and one from the treatment group [P8]) from whom sufficient cells were available from the randomization time point and in whom frequencies of viral DNA copies were sufficiently high to permit analysis by single-cell microfluidics technologies. Briefly, peripheral blood memory CD4 T cells isolated by negative immunomagnetic enrichment were stained with a total of *n* = 74 oligonucleotide-labeled monoclonal antibodies; in addition to the antibodies used in our prior work (Sun et al., 2023), we included antibodies recognizing classical and non-classical HLA class I molecules, and other markers relevant for modulating innate immune recognition. After antibody staining, cells were entered into a microfluidics analysis platform designed to perform single-cell multiplex PCRs to amplify a total of 18 HIV-1 DNA segments spanning strategically important and phylogenetically conserved regions in the HIV-1 genome; oligonucleotide tags of antibodies used for surface staining were simultaneously amplified in single cells. In total, we analyzed 111,700 memory CD4 T cells from all three study participants combined. Of those, *n* = 111,052 cells were categorized as HIV-uninfected cells (category 0 cells). *N* = 648 cells were categorized as being HIV-1-infected (category 1 cells) based on the presence of at least four HIV-containing reads each in at least two viral amplicons. Evidence for the presence of genome-intact HIV-1, defined by the detection of at least four reads each in both of the viral amplicons included in the intact proviral DNA assay (Bruner et al., 2019) (IPDA) was observed in *n* = 128 cells (category 2 cells) (Table S5). A global analysis of the phenotypic profile of all analyzed cells using uniform manifold approximation and projection (UMAP) for dimension reduction demonstrated that virally infected cells (harboring any type of proviral DNA) were widely disseminated across the entire spectrum of memory CD4 T cells, without any visible enrichment in computationally defined subpopulations of cells (Fig. 5, A and B). Of note, cells infected with genome-intact HIV-1 were also broadly disseminated across the UMAP plots. Cells harboring hypermutated viral sequences (*n* = 73, category 3 cells) also failed to display specific distinguishing phenotypic characteristics in a global UMAP analysis. A detailed investigation of the phenotypic profile of HIV-1 reservoir cells identified several markers that, in a statistical analysis adjusted for multiple comparisons, displayed significantly elevated expression on virally infected cells compared with autologous uninfected cells (Fig. 5 C); however, the fold-changes in expression intensity of such differentially expressed markers were limited and did not exceed a factor of 1.4 across all analyzed phenotypic molecules (Fig. 5 C and Table S6). Nevertheless, we noted that cells harboring genome-intact viral DNA frequently expressed significantly elevated levels of the MHC class I molecule HLA-C (adjusted P value of 8.51E−32 [category 2 versus category 0 cells]) (Fig. 5, C and D); HLA-C is a high-affinity ligand for the inhibitory killer immunoglobulin receptors (KIR) KIR2DL1, KIR2DL2, and KIR2DL3 expressed on natural killer (NK) cells (Colonna et al., 1993), implying that upregulation of HLA-C may protect virally infected cells from NK cell–mediated immune activity. Of note, HLA-C expression was highest in the study participant (P8) expressing the HLA-Cw05 allele and lower in the two other study participants who expressed HLA-Cw07 alleles (Fig. S3 B); this is consistent with known allele-specific differences in HLA-C surface expression (Kulkarni et al., 2011; Thomas et al., 2009). We also noticed a slight but significant upregulation of the non-classical HLA class I isotypes HLA-G (adjusted P value of 3.13E−35 [category 2 versus category 0 cells]) and, to a lesser extent, of HLA-F (adjusted P value of 1.56E−13 [category 2 versus category 0 cells]) on cells containing intact HIV-1 (Fig. 5, C and D). Both of these markers are involved in the negative regulation of NK cells and other innate immune cells through binding to the shared inhibitory receptors LILRB1/LILRB2 (Shiroishi et al., 2006) and to inhibitory KIR receptors (KIR2DL4 for HLA-G [Rajagopalan and Long, 1999] and KIR3DL1/KIR3DL2 for HLA-F [Goodridge et al., 2013]), although for HLA-F, binding to the activating receptors KIR2DS4 (Goodridge et al., 2013) and KIR3DS1 (Garcia-Beltran et al., 2016) has also been proposed. We failed to notice an elevated expression of HLA-E on HIV-1-infected cells in contrast to our recent analysis of cells isolated from long-term ART-treated individuals who started treatment during chronic infection (Sun et al., 2023). Interestingly, we also found evidence for upregulation of additional surface markers involved in innate immune activity on cells harboring intact HIV-1. In particular, such markers included the receptors for the anti-inflammatory and regulatory cytokines IL-10 and TGF-β, both of which have a role in regulating innate immunity (Kelly et al., 2017; Shouval et al., 2014) and can support retroviral latency (Harper et al., 2022; Samer et al., 2022), and the cytokine receptors IL-12Rβ and CCR6, which can promote Th1 and Th17 CD4 T cell polarization, respectively; persistence of HIV-1 in Th1 and Th17-polarized cells has been suggested before (Collora et al., 2022; Sun et al., 2015). Additional markers that were significantly increased on cells containing intact HIV-1 included CD127 (the receptor for the homeostatic cytokine IL-7), CD27 and CD28, the integrin CD49d, the cell adhesion molecule L-selectin (CD62L), and the glucocorticoid-induced TNFR-related protein (GITR); all of these molecules can be involved in promoting cell survival and long-term persistence of memory T cells. Markers involved in the regulation and modulation of cellular susceptibility to host effector T cell responses, such as CD200, CD39, PD-1, and PD-L1, showed minimal or no trends for enrichment on cells infected with intact HIV-1. Nevertheless, 2B4, a molecule associated with inhibitory immune effects on NK cells when expressed at high levels (Chlewicki et al., 2008), was significantly upregulated on cells carrying genome-intact HIV-1. Notably, most of the phenotypic markers that were increased in cells with intact HIV-1 in this study also tended to be upregulated in cells with defective viral DNA (Fig. S3 A), however, effects were in most cases more pronounced on cells with genome-intact viral sequences; in particular, IL-12Rβ, IL10R, TGFβR2, CD62L, CD49d, CCR6, CD127, HLA-C, and HLA-G were significantly more strongly upregulated on cells harboring intact versus defective viral DNA (Fig. 5 C). No correlation was found between the phenotypic profile for HIV-1 reservoir cells reported here for individuals who started ART during early/acute infection and corresponding data obtained from long-term ART-treated study participants who started treatment during chronic infection (Sun et al., 2023) (Fig. S3 C); this finding is consistent with our hypothesis that phenotypic profiles of HIV-1 reservoir cells are dynamically evolving over time in response to host immune activity. Collectively, these results reveal a distinct phenotypic signature of HIV-1-infected cells after short periods of ART initiated in acute infection, characterized by immune surface marker signatures that are, at least in part, suggestive of immune selection pressure mediated by innate immune responses.

## Discussion

Antiretroviral treatment initiation during acute HIV-1 infection has been associated with a higher probability of maintaining subsequent drug-free viral control and, therefore, has been viewed as a window of opportunity to manipulate viral–host interactions and long-lasting reservoir cell establishment in favor of the host. However, interactions and bidirectional engagement between host immune responses and viral reservoir cells during this specific stage of HIV-1 infection remain largely unexplored, mostly because prior investigative technologies were frequently not sufficiently sensitive to capture the unperturbed biological properties of the very small number of virally infected cells directly ex vivo. Our study sheds new light on this question by applying novel single-cell/single-genome analytic techniques to assess the viral reservoir cell pool in a well-characterized cohort of individuals who started ART in early infection and participated in a randomized-controlled human clinical trial involving therapeutic T cell vaccine administrations. We demonstrate that initiation of ART during acute/early infection is frequently associated with an efficient selection of intact proviruses integrated in gene deserts, centromeric satellite DNA, or ZNF genes; while such selection events are also visible after two decades of continuous ART in individuals who start treatment late (Lian et al., 2023), they seem to be accelerated and intensified upon early treatment initiation. Interestingly, we do not find evidence that this selection process is related to vaccine-induced, adaptive HIV-1-specific T cells; in fact, we noted that signs of immune selection tended to be more obvious in the control group of people who had not received the HIV-1 immunogens. Instead, single-cell proteogenomic profiling suggested that viral reservoir cells in acute infection are under immune selection pressure by innate immune responses; in particular, we observed subtle but significant upregulation of the HLA molecules HLA-C and HLA-G which are likely to increase resistance to killing by NK cells and, possibly, other innate immune cells. In sum, these results support the hypothesis that HIV-1 reservoir cells are vulnerable to host immune activity, specifically during the early stages of viral infection, and that innate, rather than adaptive immune responses, may be able to engage and target viral reservoir cells at early stages of infection.

While initially considered as completely resistant to host immune activity due to viral latency, it is increasingly clear that HIV-1 reservoir cells can be subject to host immune selection processes; however, their vulnerability to immune mechanisms seems to vary considerably. A series of recent studies suggested that proviral integration site location is a key determinant of the susceptibility of virally infected cells to host immune recognition and that intact proviruses able to persist long-term and clonally proliferate are typically located in highly distinct chromosomal locations that are less permissive to proviral transcription (Cole et al., 2021; Dragoni et al., 2023; Einkauf et al., 2022; Huang et al., 2021; Lian et al., 2023); these locations appear to offer a protective chromosomal environment and a “sanctuary site” for viral persistence. Using optimized technologies for the biocomputational mapping of HIV-1 integration sites, including the use of the recently-developed T2T human reference genome for alignment of chimeric viral sequencing reads, we here show that in the setting of early treatment initiation, persistence and clonal expansion of intact proviruses seem favored by integration into heterochromatin regions, including those in the upper arms of the acrocentric chromosomes. However, the intensity of selection effects varied among study persons, as we identified one individual, in whom intact proviruses persisted and expanded in a proliferation-associated gene that presumably can enable the expansion of infected cells through insertional mutagenesis (Bushman, 2020); integration into such genes may allow HIV-1-infected cells to outcompete negative host selection forces (Einkauf et al., 2022). Together, our data suggest that viral persistence in permissive chromosomal regions does not seem to be a viable option for many HIV-1-infected cells, at least not when treatment is initiated during early infection. We propose that the effective immune selection occurring throughout the treatment of acute infection may contribute to the evolution of a distinct reservoir profile that consists of intact but poorly-inducible proviruses with features of “deep latency”; such a distinct viral reservoir configuration may possibly contribute to the higher probability to maintain drug-free viral control after discontinuation of ART initiated during acute/primary infection. Future studies evaluating the longitudinal evolution of viral reservoir cells in posttreatment controllers from the beginning of ART initiation will be helpful to better define how effective immune selection of reservoir cells represents a defining element of spontaneous HIV-1 immune control.

Immune mechanisms that can drive the selection of HIV-1 reservoir cells are poorly understood at the current time and represent an area of active investigation. Our study addresses this question using samples from a randomized-controlled clinical trial evaluating a therapeutic vaccine, permitting testing the possible impact of vaccine-induced T-cell responses on HIV-1 reservoir cell selection. Our results suggest that HIV-1-specific T cell responses are unlikely to exert significant immune pressure against viral reservoir cells in early-treated individuals. In fact, longitudinal viral reservoir cell profiles did not differ significantly between recipients of therapeutic vaccines and the control group, nor did we find statistical associations between viral reservoir cells and the quantity or the functional profile of HIV-1-specific CD4 or CD8 T cell responses. This observation may be most readily explained by the low level of proviral transcription that is likely insufficient to permit adequate production of viral proteins and their processing and presentation via the MHC class I complex; of note, treatment with vorinostat failed to induce significant increases in viral transcription in the primary analysis of the RIVER study and therefore was unlikely to increase the immunological vulnerability to T cell responses in a biologically significant way. Correspondingly, recent studies by Statzu et al. (2023) also suggested that virus-specific T cells are unable to modulate or influence SIV reservoir cell evolution during primary infection in a non-human primate animal model. Whether more advanced latency-reversing agents with a higher potency for exposing viral reservoir cells to T cell–mediated immune recognition (Jones et al., 2016) may be more effective in engaging HIV-1-specific T cells for viral reservoir cell targeting remains to be determined in future clinical trials.

As an additional investigative modality, we conducted single-cell proteogenomic profiling studies to infer sources of immune selection pressure through phenotypic expression signatures on HIV-1 reservoir cells. These data revealed the upregulation of several cytokine receptors on virally infected cells, including the receptors for IL-10 and TGF-β which are released as part of the cytokine storm during primary infection and promote viral latency (Harper et al., 2022; Samer et al., 2022); proviral transcription in these cells may be more effectively repressed, translating in a lower probability of exposure to host immune recognition and a higher probability of survival. Moreover, we noted upregulation of several markers that can confer survival signals to infected cells; these observations are compatible with prior studies suggesting that persistence of virally-infected cells during suppressive ART relies on prosurvival and anti-apoptosis molecular pathways (Arandjelovic et al., 2023; Huang et al., 2018; Sun et al., 2023). Of note, our studies suggested a slight but significant elevation of HLA-C expression, and to a lesser degree, of HLA-G and HLA-F expression on cells harboring intact HIV-1; all of these markers can interact with inhibitory HLA class I receptors of innate immune cells and can be interpreted as biomarkers of innate immune selection pressure. In particular, HLA-C surface expression is tightly regulated by immunogenetic variations that influence HIV-1 disease outcomes during untreated infection (Kulkarni et al., 2011), and relatively small changes in its surface expression intensity seem to be sufficient for influencing cellular susceptibility to NK cell–mediated innate immune activity (Korner et al., 2017). Similarly, HLA-G and HLA-F surface expression intensity on target cells can modulate their vulnerability to NK cells (Chen et al., 2013; Dulberger et al., 2017). We propose that simultaneous upregulation of HLA-C, -G, and -F on viral reservoir cells during early-treated infection represents a consequence of innate immune selection that promotes preferential persistence of target cells with higher levels of resistance to cytolytic NK cells and, possibly, other innate immune cells; future studies designed to assess HLA class I expression on target cells in conjunction with their corresponding activating and inhibitory ligands on innate immune effector cells may help to further explore this hypothesis. Of note, an acceleration of HIV-1 reservoir cell selection through innate immune mechanisms has recently been reported in an interventional clinical trial (Armani-Tourret et al., 2024); moreover, inverse statistical associations between NK cells and viral reservoir cells were reported in previous observational studies (Hartana et al., 2022; Hua et al., 2018). Nevertheless, we acknowledge that the evidence supporting the role of innate immunity in driving HIV-1 reservoir cell selection in this study is based on indirect evidence from surface phenotyping studies of unperturbed HIV-1-infected cells from study participants; future studies will be necessary to further investigate and corroborate the ability of innate immune mechanisms to target and select HIV-1 reservoir cells.

In sum, our data suggest a remarkable vulnerability of HIV-1 reservoir during early-treated HIV-1 infection, highlighted by the fact that cells with intact proviruses integrated in heterochromatin seem frequently selected within a relatively short period of time. This selection process appears to be primarily driven by innate immune activity, while we failed to find evidence supporting a role for adaptive T cell responses in reservoir cell selection and evolution. Future studies designed to clarify how innate immune mechanisms can target and engage viral reservoir cells seem warranted and may help to develop more effective clinical strategies for depleting the HIV-1 reservoir cell pool.

## Materials and methods

### Cell samples

Longitudinally collected PBMC samples from participants of the RIVER clinical trial were used. Samples were collected under institutional review board protocols approved at the local study centers, as described in Fidler et al. (2020). The secondary use of sample studies at the Ragon Institute was approved by the Human Research Committee of Mass General Brigham in Boston, MA, USA. The clinical and demographical characteristics of study participants are summarized in Table S1. Study participants gave written informed consent to participate in accordance with the Declaration of Helsinki. PBMC were isolated using Ficoll-Paque density centrifugation. PBMC were viably frozen in 90–95% fetal bovine serum and 5–10% dimethyl sulfoxide and thawed at the time of analysis. For surface phenotyping assays, PBMC were subjected to negative immunomagnetic isolation of memory CD4^+^ T cells using a commercial product (#18000; Stemcell EasySep Human Memory CD4^+^ T cell Enrichment Kit) per the manufacturer’s protocol.

### Droplet digital PCR (ddPCR)

PBMCs were subjected to DNA extraction using commercial kits (#69504; Qiagen DNeasy). We amplified total HIV-1 DNA using ddPCR (Bio-Rad) and primers and probes described previously (Lee et al., 2017) (127 bp 5′LTR-gag amplicon; HXB2 coordinates 684–810). The droplets were subsequently read by the QX200 droplet reader and data were analyzed using QuantaSoft software (Bio-Rad).

### HLA class I typing

HLA typing was performed using a targeted next-generation sequencing method, as described previously (Digitale et al., 2021).

### Near full-length individual proviral sequencing (FLIP-seq)

Extracted DNA was diluted to single viral genome levels according to ddPCR results, so that 1 provirus was present in ∼20–30% of wells. DNA resulting from whole-genome amplification reactions was subjected to HIV-1 near full-genome amplification using a 1-amplicon and/or non-multiplexed 5-amplicon approach, as described before (Einkauf et al., 2019). PCR products were visualized by agarose gel electrophoresis (Quantify One and ChemiDoc MP Image Lab; Bio-Rad). All near full-length and/or 5-amplicon positive amplicons were subjected to Illumina MiSeq sequencing at the MGH DNA Core facility. Resulting short reads were de novo–assembled using Ultracycler v1.0 and aligned to HXB2 to identify large deleterious deletions (<8,000 bp of the amplicon aligned to HXB2), out-of-frame indels, premature/lethal stop codons, internal inversions, or packaging signal defects (≥15 bp insertions and/or deletions relative to HXB2), using an automated in-house pipeline written in Python programming language (https://github.com/BWH-Lichterfeld-Lab/Intactness-Pipeline) (Lee et al., 2019), consistent with prior studies (Hiener et al., 2017; Lee et al., 2017; Pinzone et al., 2019). The presence/absence of apolipoprotein B mRNA editing enzyme catalytic polypeptide-like (APOBEC) 3G/3F-associated hypermutations was determined using the Los Alamos National Laboratory (LANL) HIV Sequence Database Hypermut 2.0 (Rose and Korber, 2000) program. Viral sequences that lacked all mutations listed above were classified as “genome-intact” sequences. Sequence alignments were performed using MUSCLE (Edgar, 2004). Phylogenetic distances between sequences were examined using maximum likelihood trees in MEGA (https://www.megasoftware.net) and MAFFT (https://mafft.cbrc.jp/alignment/software) and visualized using Highlighter plots (https://www.hiv.lanl.gov/content/sequence/HIGHLIGHT/highlighter_top.html). Viral sequences were considered clonal if they had completely identical consensus sequences; single nucleotide variations in primer binding sites were not considered for clonality analysis. Clades of intact HIV-1 proviral sequences were determined using the LANL HIV Sequence Database Recombinant Identification Program (Siepel et al., 1995). Within intact HIV-1 clade B sequences, the number of sequence mutations associated with HLA class I–mediated pressure was calculated in clade-B proviruses as previously described (Carlson et al., 2012).

### Whole genome amplification

When indicated, genomic DNA from isolated PBMC were subjected to whole-genome amplification prior to full-genome amplification, as described in the MIP-Seq protocol. For this purpose, DNA in each well was subjected to MDA with phi29 polymerase (#150345; Qiagen REPLI-g Single Cell Kit), per the manufacturer’s protocol. Following this unbiased whole genome amplification (Burtt, 2011), DNA from each well was split and separately subjected to near full-length viral sequencing and integration site analysis, as described below. If necessary, a second-round MDA reaction was performed to increase the amount of available DNA.

### Integration site analysis

Integration sites associated with each viral sequence were obtained using ISLA using a protocol previously described by Wagner et al. (2014); DNA produced by whole-genome amplification was used as the template. For selected clonal sequences, viral–host junction regions were also amplified using primers annealing upstream of the integration site in host DNA and downstream of the integration site in viral DNA. The resulting PCR products were subjected to next-generation sequencing using Illumina MiSeq. MiSeq paired-end FASTQ files were demultiplexed; small reads (142 bp) were then aligned simultaneously to human reference genome GRCh38 and HIV-1 reference genome HXB2 using bwa-mem (Li and Durbin, 2009). In selected cases, the T2T human reference genome was sued for alignment. Biocomputational identification of integration sites was performed according to previously described procedures (Serrao et al., 2016; Wagner et al., 2014): Briefly, chimeric reads containing both human and HIV-1 sequences were evaluated for mapping quality based on (i) HIV-1 coordinates mapping to the terminal nucleotides of the viral genome, (ii) absolute counts of chimeric reads, (iii) depth of sequencing coverage in the host genome adjacent to the viral integration site. The final list of integration sites (Table S2) and corresponding chromosomal annotations was obtained using Ensembl (v110, https://www.ensembl.org), the UCSC Genome Browser (https://www.genome.ucsc.edu) and GENCODE (v44, https://www.gencodegenes.org). Repetitive genomic sequences harboring HIV-1 integration sites were identified using RepeatMasker (https://www.repeatmasker.org).

### Surface labeling with monoclonal antibodies

Monoclonal antibodies tagged with distinct oligonucleotides were custom-manufactured and supplied as lyophilized single reaction vials by a commercial vendor (Biolegend). Antibodies against the following surface markers were used: PD-L1 (clone 29E.2A3), CD276 (clone DCN.70), HVEM (clone122), CD155 (clone SKII.4), CD154/CD40L (clone 24-31), CCR4 (clone L291H4), PD-1 (A17188B), TIGIT (clone A15153G), CD44 (clone BJ18), CXCR3 (clone G025H7), CCR5 (clone HEK/1/85a), CCR6 (clone G034E3), CXCR5 (clone J252D4), CCR7 (clone G043H7), KLRB1/CD161 (clone HP-3G10), CTLA-4 (clone BNI3), LAG-3 (clone 11C3C65), KLRG1 (clone 14C2A07), CD95 (clone, DX2), OX40/CD134 (clone Ber-ACT35), CD57 (clone HNK-1), TIM-3 (clone F38-2E2), BTLA/CD272 (clone MIH26), CD244/2B4 (clone 2-69), IL-2R (clone TU27), CD137/4-1BB (clone 4B4-1), GITR/CD357 (clone 108-17), CD28 (clone CD28.2), CD127 (clone A019D5), IL-6R (clone UV4), HLA-E (clone 3D12), MICA/B (clone 6D4), IL-15R/CD215 (clone JM7A4), IL-21R (clone 2G1-K12), TNFR2 (clone 3G7A02), CD160 (clone BY55), LIGHT/CD258 (clone T5-39), IL-10R/CD210 (clone 3F9), TGFB-R (clone W17055E), IL-12R (clone S16020B), CD6 (clone BL-CD6), CD49d (clone 9F10), CD25 (clone BC96), CD30 (clone BY88), CD69 (clone FN50), CD45RA (clone HI100), CD38 (clone HIT2), HLA-DR (clone L243), CD4 (clone RPA-T4), CD2 (clone TS1/8), CD3 (clone UCHT1), CD62L (clone DREG-56), CD45RO (clone UCHL1). Two mouse IgG control antibodies (#400299, #400383; Biolegend), conjugated with distinct TotalSeq-D oligonucleotides, were included as isotype controls. The lyophilized antibody cocktails containing the above-mentioned antibodies were reconstituted with cell staining buffer (#420201; Biolegend). The following antibodies were spiked-in into the reconstituted antibody cocktails: CD112/Nectin-2 (clone TX31), CD273/PD-L2 (clone 24F.10C12), CD200 (clone OX-104), CD48/SLAMF2 (clone BJ40), CD40 (clone 5C3), HLA-C (clone DT-9), HLA-G (clone 87G), CD103 (clone Ber-ACT8), CD11a (clone TS2/4), CD27 (clone O323), CD278/ICOS (clone C398.4A), CD39 (clone A1), CD47 (clone CC2C6), CD54 (clone HA58), CD59 (clone p282 [H19]), b2M (clone 2M2), HLA-F (clone 3D11/HLA-F), TRAIL-R1/CD261 (clone DJR1), and TRAIL-R2/CD262 (clone DJR2-4 [7–8]). Cells were blocked with Human TruStain FcX (#422301; Biolegend) for 15 min on ice and then incubated with 50 µl of reconstituted antibody cocktail for 30 min on ice. After three washes with a prechilled cell-staining buffer, cells were filtered with a 40-μm cell Flowmi strainer (#14-100-150; Thermo Fisher Scientific), counted with an automated cell counter, and then loaded into a microfluidic cartridge for single-cell multiplex PCR assays.

### Single-cell multiplex PCR

Single-cell amplification of defined genomic DNA segments was performed using the Tapestri platform (MissionBio) according to the manufacturer’s protocol (Ruff et al., 2022) and as described in our previous work (Sun et al., 2023). Briefly, viable single cells were encapsulated into droplets with a lysis buffer and incubated for 1 h at 50°C, followed by 10 min at 80°C for heat inactivation of enzymes. Droplets containing single-cell barcoding beads (tagged with oligonucleotides carrying the cellular barcodes and custom-designed primers) were fused with encapsulated cell lysates. A panel of primers designed to amplify *n* = 18 different genomic regions in addition to two primer sets amplifying control genomic regions within the RPP30 gene on chromosome 10, as described before (Sun et al., 2023). The droplets were placed under UV light to cleave PCR primers containing unique cell barcodes from beads. To amplify the selected genomic DNA segments and the antibody oligonucleotide tags, droplets were subjected to PCR for 24 cycles with temperature gradients recommended by the manufacturer.

### Sequencing library construction

Amplification products were pooled, mixed with AMPure XP beads (#A63882; Beckman Coulter) at a ratio of 0.7, and placed in a magnetic field for separating the DNA and the protein tag libraries. The DNA library bound to AMPure beads was washed with 80% ethanol, while the supernatant containing the protein tag library was aspirated and incubated with a biotinylated oligonucleotide complementary to the 5′ end of the antibody tags, followed by magnetic isolation using streptavidin beads. For library amplification, PCRs were performed with Illumina index primers P5 and P7 on purified DNA and protein libraries, respectively, according to the manufacturer’s protocol; 12 cycles were performed for the DNA library and 18 cycles were run for the protein tag library.

### Next-generation sequencing

The DNA and protein tag libraries were run on a High Sensitivity D1000 ScreenTape instrument (5067-5584; Agilent Technologies) with the Agilent 4200 TapeStation System to evaluate DNA quality. Libraries were quantified by a fluorometer (Qubit 4.0; Invitrogen) and sequenced on Illumina next-generation sequencing platforms with a 20% spike-in of PhiX control DNA (#FC-110-3002; Illumina). DNA and protein tag libraries were sequenced separately on a NextSeq2000 instrument (Illumina), using NextSeq 2000 P3 Reagents (200 Cycles) (# 20040560; Illumina) in 2 × 100 bp paired-end runs for protein libraries and NextSeq 2000 P3 Reagents (300 Cycles) (# 20040561; Illumina) in 2 × 150 bp paired-end runs for DNA libraries.

### Bioinformatic analysis

The Tapestri pipeline (v2.0.1; MissionBio) with minor modifications was used to process the sequencing data. Briefly, for DNA library data, cutadapt (v2.5) (Martin, 2011) was used to trim 5′ and 3′ adapter sequences and extract 18 bp cell barcode sequences from Read 1. Cell barcodes that aligned to a unique barcode on a whitelist within a Hamming distance of 2 were used for downstream analysis. Using bwa (v0.7.12) (Li and Durbin, 2009), sequences were aligned to custom reference genomes built from the human genome (hg38) and patient-specific autologous HIV-1 sequences identified in prior studies. Single-cell alignments were filtered according to criteria implemented in the Tapestri pipeline, and indexed using samtools (v1.9) (Li et al., 2009). Candidate HIV-1-infected cells were determined by the CellFinder algorithm built in the Tapestri pipeline. Bcftools (v1.9) (Li, 2011) was used to call variants and generate consensus sequences. To reduce spurious alignments, viral sequencing reads were only considered valid if they covered at least 80% of the length of the reference sequence for each given amplicon. For antibody library data, cell barcodes were similarly extracted using cutadapt. For reads with valid cell barcodes, 15-bp antibody barcodes were extracted from Read 2. Antibody barcode sequences within a Hamming distance of 1 from known antibody barcodes were accepted. Candidate cells appearing in both libraries were processed for downstream analysis. Read counts for each antibody were normalized to the total read count within each cell using a centered log-ratio (CLR) transformation (Aitchison, 1989). Cut-offs for a given phenotypic marker to be considered positive were defined by marker-specific read counts >1 mean absolute deviation of the normalized median read count corresponding to unspecific IgG control antibodies. To generate a more homogenous cell population for analysis, CLR values of all candidate cells that were CD3^−^CD4^−^ (non CD4^+^ T cells) and CCR7^+^CD45RA^+^ (contaminating naive CD4^+^ T cells) were excluded.

### Dimension reduction and clustering

UMAP embeddings in two dimensions of the CLR values were done through the Monocle 3 (Cao et al., 2019) and uwot (Melville, 2020) packages with the number of principal components set to 15 for all cells, and 10 for HIV-1-infected cells only; numbers of neighbors to use during k-nearest neighbors graph construction were set to 9 and 5, respectively. All other settings were kept to the default values. The cells were clustered using the Leiden community detection algorithm through Monocle 3, with the k-near neighbors set to 500.

### Phylogenetic analysis of HIV-1

HIV-1 sequencing reads corresponding to each of the 18 HIV-1 amplification products were aligned to the reference HIV-1 genome HXB2, to autologous intact HIV-1 sequences. The presence/absence of APOBEC-3G/3F-associated hypermutations was determined using the LANL HIV Sequence Database Hypermut 2.0 program (Rose and Korber, 2000). Proviruses were classified into three different categories according to the following criteria: category 1 (any provirus): at least 20 total valid viral sequencing reads with at least 4 reads in at least 2 different HIV-1 amplicons each; category 2 (enriched for intact proviruses): a total of at least 20 viral sequencing reads with at least 4 sequencing reads from amplicons 2 and 16 (corresponding to IPDA amplicons) each or at least 4 reads from at least 15 amplification products each; all viral sequencing reads in category 2 had to be without evidence of statistically significant hypermutation; category 3 (hypermutated proviruses): proviral sequences from category 1 that displayed statistically significant sequence hypermutations (false discovery rate [FDR]-adjusted P < 0.05). Cells with HIV-1 sequencing reads that did not meet any of the above-mentioned criteria were excluded from the analysis. Category 0 cells were defined by the complete absence of sequencing reads corresponding to HIV-1.

### Statistics

Data are presented as pie charts, bar charts, scatter plots with individual values, UMAP plots, heatmaps, or volcano plots. Enrichment ratios between two cell populations for each marker were calculated as the ratio of the proportion of marker-positive cells in the first population divided by the proportion of marker-positive cells in the second population. Sensitivity values were calculated by dividing the number of marker-positive cells in each category of cells by the total number of cells in the respective cell category. A bootstrapped dataset was constructed by resampling equal numbers of cells from each cell category from each participant, while keeping the total number of cells from each cell category equal between the bootstrapped and raw datasets. Differences were tested for statistical significance using Fisher’s exact test, chi-square test, Wilcoxon matched-pairs signed rank test, Friedman test followed by Dunn’s multiple comparisons test, Mann–Whitney *U* test, or two-sided Kruskal–Wallis nonparametric test, as appropriate. P values of <0.05 were considered significant; FDR correction was performed using the Benjamini-Hochberg method (Benjamini and Hochberg, 1995). Analyses were performed using Prism (GraphPad Software, Inc.) and R (R Core Team, 2019). Figures were generated using Adobe Illustrator.

### Online supplemental material

Fig. S1 shows a detailed analysis of HIV-1 proviral sequences from RIVER participants. Fig. S2 summarizes the clonality and chromosomal locations of all defective proviral sequences from participants 3 and 8 and an analysis of the chromosomal distance between integration sites of all intact proviruses from the RIVER study to most proximal host TSSs. Fig. S3 shows a comprehensive analysis of phenotypic characteristics of HIV-1 reservoir cells. Table S1 summarizes the demographical, clinical, and immunogenetic data of study participants. Table S2 shows statistical associations between proviral sequences and HIV-specific T cell responses. Table S3 lists all the integration sites in all RIVER study participants. Table S4 indicates statistical correlations between HIV-1-specific T cell responses and frequencies of intact HIV-1 proviruses integrated in repressive chromatin. Table S5 lists the cell numbers analyzed by PheP-Seq from three RIVER participants. Table S6 shows the relative enrichment ratios for all phenotypic markers in indicated HIV-1 reservoir cell categories analyzed by PheP-Seq.

## Supplementary Material

Table S1 shows demographical, clinical, and immunogenetic data of study participants.

Table S2 shows statistical associations between proviral sequences and HIV-1-specific T cell responses.

Table S3 lists of proviral integration sites of intact and defective proviruses in all RIVER study participants.

Table S4 shows statistical associations between intact proviral sequences integrated in repressive chromatin and HIV-1-specific T cell responses.

Table S5 shows cell numbers analyzed by PheP-Seq from three study participants in each cell category.

Table S6 shows relative enrichment ratios for all phenotypic markers in indicated HIV-1 reservoir cell categories analyzed by PheP-Seq.

## Data Availability

The integration site data and enrichment ratios of phenotypic markers obtained by PheP-Seq are provided in Tables S3 and S6, respectively. Owing to study participant confidentiality concerns, viral sequencing data cannot be publicly released but will be made available to investigators upon reasonable request and after signing a data-sharing agreement.
