# Supplementary material for: Footprints of innate immune activity during HIV-1 reservoir cell evolution in early-treated infection
Source: J Exp Med. 2024 Oct 28;221(11):e20241091. doi: 10.1084/jem.20241091 (PMC11519379; doi:10.1084/jem.20241091)
Supplement: Table S1 — shows demographical, clinical, and immunogenetic data of study participants. [file JEM_20241091_TableS1.docx]

Table S1. Demographical, clinical, and immunogenetic characteristics of all study participants

| Participant | RIVER group | Gender | Ethnicity | Weeks since PHI  diagnosis (at enrollment) | Age (years) at  randomization | CD4 T cell count at  randomization  (cells/mm3) | HIV-RNA at  randomization sign | HIV-RNA at  randomization  (copies/mL) | HLA-A1 | HLA-A2 | HLA-B1 | HLA-B2 | HLA-Cw1 | HLA-Cw2 |
| --- | --- | --- | --- | --- | --- | --- | --- | --- | --- | --- | --- | --- | --- | --- |
| P1 | Control | Male | White | 1 | 31 | 758 | = | 21 | 03:01 | 24:02 | 07:02 | 07:02 | 07:02 | 07:02 |
| P2 | Control | Male | White | 3 | 35 | 675 | < | 20 | 02:01 | 03:01 | 44:02 | 49:01 | 07:01 | 07:04/11 |
| P3 | Control | Male | White | 2 | 40 | 712 | = | 37 | 01:01/04N | 11:01 | 07:02 | 49:01 | 07:01 | 07:02 |
| P4 | Control | Male | Mixed ethnic group | 2 | 25 | 644 | < | 20 | 02:01 | 24:02 | 13:01 | 44:03 | 04:01 | 07:02 |
| P5 | Control | Male | White | 4 | 26 | 729 | < | 20 | 11:01 | 68:01 | 14:01 | 18:01 | 07:01 | 08:02 |
| P6 | Treatment | Male | White | 69* | 46 | 724 | < | 20 | 01:01/04N | 02:01 | 08:01 | 27:05 | 02:02 | 07:01 |
| P7 | Treatment | Male | White | 3 | 40 | 794 | = | 24 | 01:01/04N | 02:01 | 08:01 | 44:02 | 05:01/03 | 07:01 |
| P8 | Treatment | Male | White | 2 | 44 | 688 | = | 22 | 30:01 | 30:02 | 18:01 | 35:01 | 04:01 | 05:01/03 |
| P9 | Treatment | Male | White | 2 | 28 | 1051 | < | 20 | 02:01 | 02:05 | 50:01 | 58:01 | 05:01/03 | 07:01 |
| P10 | Treatment | Male | Hispanic/Latino | 4 | 52 | 579 | < | 20 | 03:01 | 23:01 | 07:02 | 40:01 | 03:04 | 07:02 |

*This participant was enrolled into the RIVER study at week 69 after diagnosis, but ART treatment was initiated during acute infection

| Cohort | Participant numbers | Male% | Age* | CD4 T cell count/μl* | Recorded duration of undetectable viremia (years)* | Time since diagnosis (years)* |
| --- | --- | --- | --- | --- | --- | --- |
| EC | 72 | 81.9 | 57 (31-75) | 882 (450-2282) | 8.8 (1-24.2) | 17.7 (1.3-34.4) |
| ART | 43 | 69.8 | 55 (34-73) | 727 (316-1657) | 8 (1-19) | 18 (1-35) |

*Values are median (min-max).
