# Supplementary material for: Footprints of innate immune activity during HIV-1 reservoir cell evolution in early-treated infection
Source: J Exp Med. 2024 Oct 28;221(11):e20241091. doi: 10.1084/jem.20241091 (PMC11519379; doi:10.1084/jem.20241091)
Supplement: Table S2 — shows statistical associations between proviral sequences and HIV-1-specific T cell responses. [file JEM_20241091_TableS2.docx]

Table S2. Correlations between HIV-1-specific T cell responses and frequencies of intact/total HIV-1 proviruses

| Virology | T cell immune response | Immune parameter | Spearman correlation (Randomization) | | Spearman correlation (12 Weeks*) | |
| --- | --- | --- | --- | --- | --- | --- |
|  |  |  | r | p-value | r | p-value |
| Intact HIV-1 DNA copies/million PBMCs | CD4+ T-cell responses | %CD107a+ | -0.09559 | 0.794 | 0.07976 | 0.8355 |
|  |  | %CD154+ | -0.06667 | 0.8651 | 0.006061 | >0.9999 |
|  |  | %IFNγ+ | -0.4073 | 0.2413 | 0.1879 | 0.6073 |
|  |  | %IL2+ | -0.1824 | 0.6128 | -0.1636 | 0.6567 |
|  |  | % TNFα+ | 0.3091 | 0.3869 | -0.07879 | 0.8382 |
|  |  | %CD154+IFNγ+IL2+TNFa+ | 0.06253 | 0.8657 | 0.02432 | 0.9528 |
|  |  | %CD154+IFNγ+IL2+TNFa- | 0.2901 | 0.6 | -0.4101 | 0.2417 |
|  |  | %CD154+IFNγ+IL2-TNFa+ | 0.04295 | 0.9172 | 0.3963 | 0.2559 |
|  |  | %CD154+IFNγ+IL2-TNFa- | -0.07976 | 0.8355 | -0.04377 | 0.9081 |
|  |  | %CD154+IFNγ+ | 0.1288 | 0.7292 | -0.07976 | 0.8355 |
| Intact HIV-1 DNA copies/million PBMCs | CD8+ T-cell responses | % CD107a+ | -0.2371 | 0.506 | 0.3161 | 0.3707 |
|  |  | % CD154+ | -0.4724 | 0.1719 | 0.07879 | 0.8382 |
|  |  | % IFNγ+ | 0.2364 | 0.5135 | 0.3939 | 0.2632 |
|  |  | % IL2+ | -0.03127 | 0.9367 | 0.2371 | 0.506 |
|  |  | % TNFα+ | 0.4985 | 0.1456 | 0.3697 | 0.2957 |
|  |  | %CD107a+IFNγ+TNFa+ | 0.1824 | 0.6128 | 0.1152 | 0.7589 |
|  |  | %CD107a+IFNγ+TNFa- | 0.3333 | 0.3487 | 0.0303 | 0.946 |
|  |  | %CD107a+IFNγ-TNFa+ | 0.2128 | 0.5519 | -0.2364 | 0.5135 |
|  |  | %CD107a+IFNγ-TNFa- | -0.3951 | 0.2567 | -0.09203 | 0.8088 |
|  |  | %CD107a+IFNγ+ | 0.2432 | 0.4957 | 0.2 | 0.5837 |
| Total HIV-1 DNA copies/million PBMCs | CD4+ T-cell responses | %CD107a+ | 0.05462 | 0.8849 | 0.497 | 0.1491 |
|  |  | %CD154+ | -0.1273 | 0.733 | -0.1636 | 0.6567 |
|  |  | %IFNγ+ | -0.5471 | 0.1064 | 0.4303 | 0.2182 |
|  |  | %IL2+ | -0.1033 | 0.7769 | -0.2606 | 0.4697 |
|  |  | % TNFα+ | 0.2121 | 0.5603 | -0.2121 | 0.5603 |
|  |  | %CD154+IFNγ+IL2+TNFa+ | 0.08129 | 0.8248 | 0.08511 | 0.8177 |
|  |  | %CD154+IFNγ+IL2+TNFa- | 0.4062 | 0.4 | -0.3505 | 0.3333 |
|  |  | %CD154+IFNγ+IL2-TNFa+ | 0.1043 | 0.7815 | 0.2744 | 0.4399 |
|  |  | %CD154+IFNγ+IL2-TNFa- | 0.01841 | 0.9728 | 0.419 | 0.2263 |
|  |  | %CD154+IFNγ+ | 0.1534 | 0.6774 | 0.07976 | 0.8355 |
| Total HIV-1 DNA copies/million PBMCs | CD8+ T-cell responses | % CD107a+ | -0.2675 | 0.452 | 0.2614 | 0.4616 |
|  |  | % CD154+ | -0.4233 | 0.2256 | -0.04242 | 0.9184 |
|  |  | % IFNγ+ | 0.2364 | 0.5135 | 0.0303 | 0.946 |
|  |  | % IL2+ | 0.1 | 0.7825 | -0.2918 | 0.4106 |
|  |  | % TNFα+ | 0.5471 | 0.1064 | 0.04242 | 0.9184 |
|  |  | %CD107a+IFNγ+TNFa+ | 0.1945 | 0.5885 | -0.1152 | 0.7589 |
|  |  | %CD107a+IFNγ+TNFa- | 0.2606 | 0.4697 | -0.3455 | 0.3304 |
|  |  | %CD107a+IFNγ-TNFa+ | 0.3161 | 0.3707 | 0.05455 | 0.8916 |
|  |  | %CD107a+IFNγ-TNFa- | -0.2918 | 0.4106 | 0.1902 | 0.602 |
|  |  | %CD107a+IFNγ+ | 0.2188 | 0.5414 | -0.07879 | 0.8382 |
