# Supplementary material for: Footprints of innate immune activity during HIV-1 reservoir cell evolution in early-treated infection
Source: J Exp Med. 2024 Oct 28;221(11):e20241091. doi: 10.1084/jem.20241091 (PMC11519379; doi:10.1084/jem.20241091)
Supplement: Table S4 — shows statistical associations between intact proviral sequences integrated in repressive chromatin and HIV-1-specific T cell responses. [file JEM_20241091_TableS4.docx]

Table S4. Correlations between HIV-1-specific T cell responses and frequencies of intact HIV-1 proviruses integrated in repressive chromatin

| Virology | T cell immune response | Immune parameter | Spearman correlation (Randomization) | | Spearman correlation (12 Weeks*) | |
| --- | --- | --- | --- | --- | --- | --- |
|  |  |  | r | p-value | r | p-value |
| Intact HIV-1 DNA copies in  repressive chromatin/million  PBMCs | CD4+ T-cell responses | %CD107a+ | -0.006828 | 0.9925 | 0.06749 | 0.8622 |
|  |  | %CD154+ | -0.06667 | 0.8651 | 0.2727 | 0.4483 |
|  |  | %IFNγ+ | -0.4134 | 0.2349 | 0.6 | 0.0734 |
|  |  | %IL2+ | -0.1885 | 0.5993 | 0.1879 | 0.6073 |
|  |  | % TNFα+ | 0.1515 | 0.6821 | 0.3939 | 0.2632 |
|  |  | %CD154+IFNg+IL2+TNFa+ | 0.1188 | 0.7429 | 0.3769 | 0.2816 |
|  |  | %CD154+IFNg+IL2+TNFa- | 0.4062 | 0.4 | 0.007457 | >0.9999 |
|  |  | %CD154+IFNg+IL2-TNFa+ | 0.04295 | 0.9172 | 0.5976 | 0.0731 |
|  |  | %CD154+IFNg+IL2-TNFa- | -0.006135 | >0.9999 | 0.2251 | 0.5279 |
|  |  | %CD154+IFNg+ | 0.1411 | 0.7026 | 0.2393 | 0.5077 |
| Intact HIV-1 DNA copies in  repressive chromatin/million  PBMCs | CD8+ T-cell responses | % CD107a+ | -0.4012 | 0.2499 | -0.07903 | 0.8298 |
|  |  | % CD154+ | -0.4356 | 0.2108 | 0.01818 | 0.973 |
|  |  | % IFNγ+ | 0.07879 | 0.8382 | 0.06667 | 0.8651 |
|  |  | % IL2+ | 0.01876 | 0.9651 | -0.1033 | 0.7769 |
|  |  | % TNFα+ | 0.4438 | 0.1989 | 0.09091 | 0.8113 |
|  |  | %CD107a+IFNg+TNFa+ | 0.0304 | 0.9382 | 0.01818 | 0.973 |
|  |  | %CD107a+IFNg+TNFa- | 0.1394 | 0.7072 | -0.1515 | 0.6821 |
|  |  | %CD107a+IFNg-TNFa+ | 0.1155 | 0.7506 | 0.1515 | 0.6821 |
|  |  | %CD107a+IFNg-TNFa- | -0.462 | 0.1804 | -0.3988 | 0.2556 |
|  |  | %CD107a+IFNg+ | 0.06687 | 0.8568 | -0.01818 | 0.973 |
