# Supplementary material for: Footprints of innate immune activity during HIV-1 reservoir cell evolution in early-treated infection
Source: J Exp Med. 2024 Oct 28;221(11):e20241091. doi: 10.1084/jem.20241091 (PMC11519379; doi:10.1084/jem.20241091)
Supplement: Table S5 — shows cell numbers analyzed by PheP-Seq from three study participants in each cell category. [file JEM_20241091_TableS5.docx]

Table S5. Cell numbers analyzed by PheP-Seq from each study participant in each cell category

| Participant | Timepoint | Total cells  analyzed | Category 0 | Category 1 | Category 2 | Category 3 |
| --- | --- | --- | --- | --- | --- | --- |
|  |  |  | HIV-uninfected cells | Total HIV-1-infected cells | Cells with intact HIV-1  proviruses | Cells with hypermutated  HIV-1 |
| P1 | Randomization | 73,942 | 73,641 | 301 | 12 | 12 |
| P3 | Randomization | 27,593 | 27,401 | 192 | 55 | 55 |
| P8 | Randomization | 10,165 | 10,010 | 155 | 61 | 6 |
| Total |  | 111,700 | 111,052 | 648 | 128 | 73 |
